# Keeping Balance Between Genetic Stability and Plasticity at the Telomere and Subtelomere of *Trypanosoma brucei*

**DOI:** 10.3389/fcell.2021.699639

**Published:** 2021-07-05

**Authors:** Bibo Li

**Affiliations:** ^1^Center for Gene Regulation in Health and Disease, Department of Biological, Geological, and Environmental Sciences, College of Sciences and Health Professions, Cleveland State University, Cleveland, OH, United States; ^2^Case Comprehensive Cancer Center, Case Western Reserve University, Cleveland, OH, United States; ^3^Department of Inflammation and Immunity, Lerner Research Institute, Cleveland Clinic, Cleveland, OH, United States; ^4^Center for RNA Science and Therapeutics, Case Western Reserve University, Cleveland, OH, United States

**Keywords:** telomere, *Trypanosoma brucei*, genome stability, TRF, RAP1

## Abstract

Telomeres, the nucleoprotein complexes at chromosome ends, are well-known for their essential roles in genome integrity and chromosome stability. Yet, telomeres and subtelomeres are frequently less stable than chromosome internal regions. Many subtelomeric genes are important for responding to environmental cues, and subtelomeric instability can facilitate organismal adaptation to extracellular changes, which is a common theme in a number of microbial pathogens. In this review, I will focus on the delicate and important balance between stability and plasticity at telomeres and subtelomeres of a kinetoplastid parasite, *Trypanosoma brucei*, which causes human African trypanosomiasis and undergoes antigenic variation to evade the host immune response. I will summarize the current understanding about *T. brucei* telomere protein complex, the telomeric transcript, and telomeric R-loops, focusing on their roles in maintaining telomere and subtelomere stability and integrity. The similarities and differences in functions and underlying mechanisms of *T. brucei* telomere factors will be compared with those in human and yeast cells.

## Introduction

As eukaryotic cells have evolved to have linear chromosomes, so has telomere evolved to play a key role in maintaining genome integrity and chromosome stability. Telomeres are the nucleoprotein complexes at linear chromosome ends. The telomere sequence, structure, and telomere-associated proteins play essential roles in proper telomere length maintenance, chromosome end protection, and regulation of subtelomeric gene expression (Ottaviani et al., 2008; Sobinoff and Pickett, 2017; de Lange, 2018; Laberthonnière et al., 2019). Nevertheless, recent studies in unicellular protozoan parasites and fungi suggest that subtle telomere and subtelomere instability can be beneficial for individual organism to adapt to challenging growth environment in the short term and can contribute to species evolution in the long term. In this review, I will first briefly summarize key telomere functions, then describe the relationship between telomere stability and antigenic variation in a protozoan parasite *Trypanosoma brucei*, focusing on similar and different challenges faced by *T. brucei* telomere proteins and those in mammalian and yeast cells. Last I will discuss potential benefit of limited telomere stability, especially in a number of microbial pathogens.

## Telomeres Are Essential for Genome Stability

Telomeres in many eukaryotes consist of simple repetitive TG-rich sequences (Podlevsky et al., 2008). All vertebrates, a small number of insects, a few species of plants and amoeba, and several kinetoplastids including *T. brucei* and *Leishmania* have telomeres with a sequence of perfect TTAGGG repeats (Podlevsky et al., 2008). Telomeres are mostly double stranded, but a terminal single-stranded overhang structure has been observed in many organisms, including vertebrates (150–400 nt long) and yeasts (12–40 nt long) (Wellinger et al., 1993; Makarov et al., 1997). There is also a single-stranded TG-rich 3′ overhang structure at the end of *T. brucei* telomere, although it appears to be very short (∼12 nt long) (Sandhu and Li, 2011, Sandhu and Li, 2017). This terminal G-overhang can invade the duplex telomere region and form the T-loop structure, which has been observed in human, mouse, chicken, *T. brucei*, ciliates, common garden pea, *Caenorhabditis elegans*, and *Kluyveromyces lactis* (Griffith et al., 1999; Murti and Prescott, 1999; Munoz-Jordan et al., 2001; Cesare et al., 2003, 2008; Nikitina and Woodcock, 2004; Raices et al., 2008). The T-loop structure buries the telomere G-overhang, which suppresses ATM activation at mammalian telomeres (Van Ly et al., 2018). In addition, telomere binding proteins can also help protect the telomere by inhibiting Non-Homologous End Joining (NHEJ) (Smogorzewska et al., 2002; Celli and de Lange, 2005; Deng et al., 2009a; Pardo and Marcand, 2005), Homologous Recombination (HR) (Wang et al., 2004; Guo et al., 2007; Sfeir et al., 2010; Chen et al., 2011), and Microhomology-Mediated End Joining (MMEJ) (Rai et al., 2010; Sfeir and de Lange, 2012) at the telomere.

Naked telomere DNA is not only vulnerable to nucleolytic degradation but also resembles a DNA double-strand break (DSB) product. Therefore, without the protection from telomere associated proteins, DNA damage response machinery is recruited to the telomere and repair processes are attempted. In fact, it is well known that removal of key telomere proteins results in chromosomes end-to-end fusions in mammalian and yeast cells (van Steensel et al., 1998; Ferreira and Cooper, 2001; Celli and de Lange, 2005; Pardo and Marcand, 2005). The resulting dicentric chromosomes can initiate the so-called breakage-fusion-bridge (BFB) cycle (de Lange, 2018): when dicentric chromosomes are pulled to opposite poles of the dividing cell, anaphase bridges form, which is frequently followed by another round of chromosome breaks and subsequent end-fusions. BFB is a severe genome instability factor and can induce loss of heterozygosity, non-reciprocal translocations, and gene amplification (Maciejowski and de Lange, 2017). In human cells, anaphase bridges can be resolved by TREX1, a cytoplasmic 3′ exonuclease, to form single-stranded DNA, which can eventually result in chromothripsis (a process where a chromosome region is broken in a single step into many fragments followed by haphazard repair) (Maciejowski et al., 2015). In addition, APOBEC- (apolipoprotein B mRNA editing enzyme, catalytic polypeptide-like) mediated kataegis (clustered C > T and C > G changes at TpC dinucleotides) can occur with chromothripsis (Maciejowski et al., 2015). Importantly, chromothripsis and kataegis have been observed in many tumor types (Cleal and Baird, 2020), and chromoanagenesis (including chromothripsis, chromoplexy, and chromoanasynthesis) has been recognized as an important mechanism of genome instability that can contribute to tumorigenesis (Cleal and Baird, 2020; Pellestor et al., 2021). Therefore, proper telomere protection is critical for genome integrity and chromosome stability, and a key function of the telomere associated factors is to prevent the natural chromosome ends from being recognized as DNA damage sites (de Lange, 2018).

Chromosome end protection relies on a number of telomere proteins to be loaded onto the telomere DNA directly through DNA binding activities or indirectly through protein-protein interactions. Therefore, telomere DNA serves as a docking site for the telomere binding proteins and proper telomere maintenance is a pre-requisite for a stable telomere. In most eukaryotes, the 3′ end of the G-rich telomere strand can be extended by telomerase, a specialized reverse transcriptase, through *de novo* DNA synthesis, which relies on its intrinsic RNA to provide the template sequence (Greider and Blackburn, 1985, 1987; Shay and Wright, 2019). With the help from the CST telomere complex (CTC1/STN1/TEN1 in mammals and Cdc13/Stn1/Ten1 in budding yeast), the C-rich telomere strand can be subsequently filled-in by primase-polymerase alpha (normally involved in lagging strand synthesis) (Feng et al., 2017; Stewart et al., 2018). The telomerase activity counteracts the “end replication problem” due to the inability of conventional DNA polymerases to fully replicate the ends of linear DNA molecules (Greider and Blackburn, 1987, 1989). In telomerase-negative cells, DNA recombination [including the break-induced replication (BIR)], and rolling circle DNA replication can serve as mechanisms to amplify telomere and subtelomere sequences, achieving the goal of telomere maintenance (Zhang and Zou, 2020).

In many organisms including *T. brucei*, both budding and fission yeasts, and human cells, telomeres form a heterochromatic structure that exerts a repressive effect on transcription of genes located at subtelomeric regions (Gottschling et al., 1990; Nimmo et al., 1994; Horn and Cross, 1997; Baur et al., 2001; Ottaviani et al., 2008; Yang et al., 2009; Tennen et al., 2011; Pandya et al., 2013; Robin et al., 2014; Laberthonnière et al., 2019). This repressive effect is position-dependent, where in general stronger effects are observed for genes located closer to the telomere, hence the name telomere position effect or telomeric silencing (Renauld et al., 1993). On the other hand, at least at some chromosome ends, the telomere sequence is transcribed into a long, non-coding RNA called TElomere Repeat-containing RNA (TERRA) in a number of organisms including *T. brucei* (Rudenko and Van der Ploeg, 1989; Damasceno et al., 2017; Nanavaty et al., 2017; Saha et al., 2019, 2021), several other kinetoplastids and *Plasmodium falciparum* (Rudenko and Van der Ploeg, 1989; Damasceno et al., 2017; Morea et al., 2021), human (Azzalin et al., 2007), mouse (Schoeftner and Blasco, 2008), fission (Bah et al., 2012) and budding yeasts (Luke et al., 2008), and birds (Solovei et al., 1994). TERRA exhibits a propensity to form an R-loop structure with the telomere DNA (Toubiana and Selig, 2018). Both TERRA and telomeric R-loop have been shown to regulate telomerase-dependent and recombination-mediated telomere maintenance and also play a role in chromosome end protection (Toubiana and Selig, 2018).

## Maintaining Telomere/Subteloemere Stability and Antigenic Variation in *Trypanosoma brucei*

*Trypanosoma brucei* is a protozoan parasite that belongs to the Euglenozoa phylum and the Kinetoplastea class and diverged from the mammals in the evolutionary tree more than 500 million years ago. *T. brucei* has linear chromosomes (van der Ploeg et al., 1984). The *T. brucei* telomere complex is also essential for maintaining genome stability in this unicellular organism (Li et al., 2005; Jehi et al., 2014a, b; Nanavaty et al., 2017; Afrin et al., 2020a), although the detailed underlying mechanisms are not exactly the same as that in mammalian and yeast cells (see below). Interestingly, *T. brucei* harbors important virulence genes encoding its major surface antigen at subtelomeres (de Lange and Borst, 1982; Hertz-Fowler et al., 2008; Müller et al., 2018), and the telomere and subtelomere stability has been shown to influence the parasite’s pathogenesis mechanism (Boothroyd et al., 2009; Hovel-Miner et al., 2012; Jehi et al., 2014a, b; Nanavaty et al., 2017; Afrin et al., 2020a). A better understanding of how these parasites maintain their genome stability and how they evade the host immune response will help their eventual elimination.

### Antigenic Variation in *T. brucei*

*Trypanosoma brucei* causes human African trypanosomiasis (HAT). Its close relatives, *Trypanosoma cruzi* and *Leishmania*, also cause debilitating Chagas disease and leishmaniasis, respectively, in humans. These kinetoplastids are important human parasites that collectively affect more than 10 million people world-wide (WHO, 2015). However, few drugs are available to treat these diseases effectively and safely with easy administering. In addition, drug resistance cases have been observed (WHO, 2015).

**FIGURE 1:**
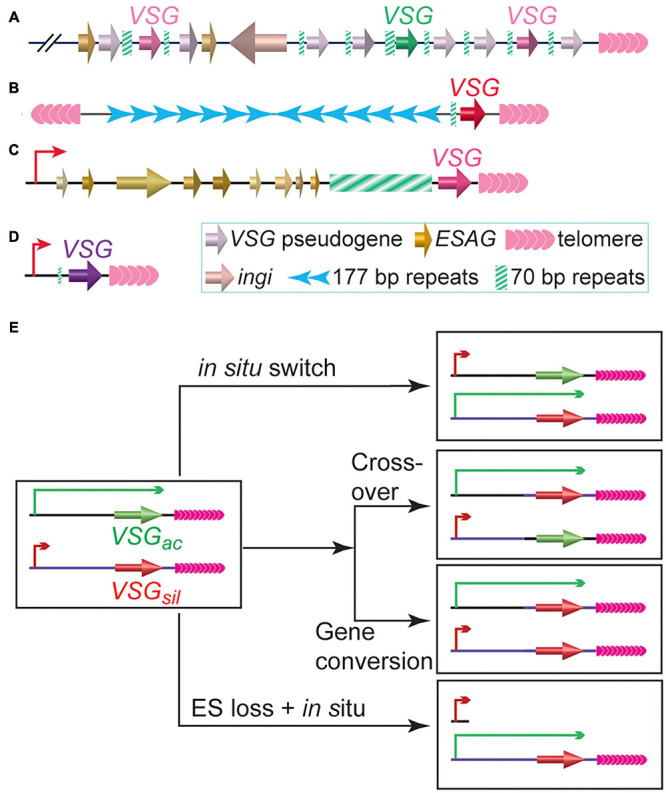
*Trypanosoma brucei* has a large *VSG* gene pool. **(A)** A representative subtelomeric *VSG* gene array. **(B)** A typical minichromosome with a subtelomeric *VSG* gene. **(C)** A representative bloodstream form VSG expression site, which is a polycistronic transcription unit and can be expressed while *T. brucei* proliferates in its mammalian host. **(D)** A representative metacyclic VSG expression site, which is a monocistronic transcription unit and can be expressed while *T. brucei* resides in the salivary gland of its insect vector. **(E)** Major VSG switching pathways. *VSG*_*ac*_ stands for the originally active *VSG*. *VSG*_*sil*_ stands for an originally silent *VSG*.

While proliferating in the extra-cellular space of its mammalian host, bloodstream form (BF) *T. brucei* expresses variant surface glycoprotein, VSG, as its major surface antigen. ∼10 million VSG proteins are packed densely on the surface of each *T. brucei* cell, masking a number of invariant surface molecules from the host immune surveillance (Morrison et al., 2009). Although *T. brucei* has a large *VSG* gene pool (>2,500 *VSG* genes and pseudogenes, Figures 1A–D) (Cross et al., 2014), VSGs are monoallelically expressed exclusively from subtelomeric polycistronic transcription units (PTUs) called VSG expression sites (ESs) (Hertz-Fowler et al., 2008; Müller et al., 2018). Each ES typically contains a single functional *VSG* as the last gene, which is flanked by upstream 70 bp repeats and downstream telomere repeats (Hertz-Fowler et al., 2008). To evade the host immune response, *T. brucei* regularly expresses immunologically distinct VSGs on the cell surface (Barry and McCulloch, 2001), although VSG switching can happen without any immune selection (Doyle et al., 1980; Myler et al., 1985). VSG switching is sometimes a transcriptional switch (*in situ*) but frequently mediated by DNA recombination (Myler et al., 1984b), where a previously silent *VSG* gene is recombined into the active ES to replace the originally active *VSG* (Figure 1E). In gene conversion (GC) events, the originally active *VSG* is lost and the donor *VSG* is duplicated (Robinson et al., 1999), while in reciprocal crossover (CO) events, the originally active and silent *VSGs* simply exchange places without any loss of genetic information (Rudenko et al., 1996; Figure 1E). Since *VSG* 3′ UTRs contain a common 14 nt sequence (Cross et al., 2014; Ridewood et al., 2017), the *VSG* 3′UTR (sometimes together with the downstream telomere sequence) and the 70 bp repeat located upstream of nearly all *VSG* genes can provide homologous sequences for efficient DNA recombination (Sima et al., 2019), and the DNA recombination-mediated VSG switching has been observed to occur more frequently than *in situ* switching in many studies (Cross et al., 1998; Robinson et al., 1999; Boothroyd et al., 2009; Kim and Cross, 2010, 2011; Hovel-Miner et al., 2012; Glover et al., 2013; Jehi et al., 2014a, b; Nanavaty et al., 2017). Many proteins involved in DNA replication, recombination, and DNA damage repair are important for VSG switching (McCulloch et al., 2015). HR can be efficiently initiated with DSBs (Haber, 2018). Indeed, introducing a DSB at the active *VSG* vicinity can induce a 250-fold higher VSG switching rate (Boothroyd et al., 2009; Glover et al., 2013). This is likely why the extremely short telomere downstream of the active *VSG* in telomerase negative cells (Hovel-Miner et al., 2012) and depletion of several telomere proteins that diminish telomere integrity/stability lead to increased VSG switching frequencies (Jehi et al., 2014a, b, 2016; Nanavaty et al., 2017; Afrin et al., 2020a).

### *T. brucei* Telomeres Face Different Telomere Instability Threats Than Those in Mammalian and Yeast Cells

Major pathways for DSB repair include HR, NHEJ, and MMEJ (Ceccaldi et al., 2016). HR has a high fidelity to repair DSBs, seldom introducing mutations (Haber, 2018). However, HR requires a homologous sequence as the repair template, which is usually only feasible in the late S/G2 phase during vegetative growth (Haber, 2018). NHEJ does not require a homologous sequence and can repair DSBs efficiently during G1 (Chang et al., 2017). MMEJ requires very short sequence homology (one or more bps) but frequently introduces insertion-deletions or even translocations and other chromosome rearrangements and is considered an error-prone DNA repair pathway (Sfeir and Symington, 2015; Seol et al., 2018). MMEJ events have been detected in *T. brucei* (Burton et al., 2007; Glover et al., 2008, 2011), although whether MMEJ can mediate VSG switching or cause chromosome end fusions in *T. brucei* is currently unknown.

In mammalian cells, Shelterin [the core telomere protein complex including TRF1 (Chong et al., 1995), TRF2 (Bilaud et al., 1997; Broccoli et al., 1997b), Repressor Activator Protein 1 (RAP1) (Li et al., 2000), TIN2 (Kim et al., 1999), POT1 (Baumann and Cech, 2001; Loayza and de Lange, 2003), and TPP1 (Houghtaling et al., 2004; Liu et al., 2004; Ye et al., 2004)] not only inhibits ATM and ATR activation (Karlseder et al., 1999; Celli and de Lange, 2005; Wu et al., 2006; Denchi and de Lange, 2007; Guo et al., 2007; Hockemeyer et al., 2007; Gong and de Lange, 2010; Takai et al., 2011; Frescas and de Lange, 2014; Van Ly et al., 2018), suppresses NHEJ (Smogorzewska et al., 2002; Doksani et al., 2013; Arnoult et al., 2017; de Lange, 2018) and HR (Wang et al., 2004; Wu et al., 2006; Palm et al., 2009; Sfeir et al., 2010) at the telomere, but also prevents nucleolytic degradation (Sfeir and de Lange, 2012; Lottersberger et al., 2013; Kibe et al., 2016) and suppresses MMEJ (Rai et al., 2010; Sfeir and de Lange, 2012; Mateos-Gomez et al., 2015). In addition, TERRA is transcribed by RNA Polymerase II at many telomeres from subtelomeric CpG islands-containing promoters in human cells (Nergadze et al., 2009; Deng et al., 2012; Porro et al., 2014a; Diman et al., 2016; Feretzaki and Lingner, 2017). Telomeric R-loops are detectable in human primary cells (Shiromoto et al., 2021) and HeLa cells (Feretzaki et al., 2020), while ALT cancer cells and ICF syndrome cells, in which TERRA is transcribed at a higher than normal level (Yehezkel et al., 2008; Arora et al., 2014), appear to have more telomeric R-loops (Arora et al., 2014; Min et al., 2017; Nguyen et al., 2017; Sagie et al., 2017). R-loops have been shown to be a genome instability factor (García-Muse and Aguilera, 2019; Brambati et al., 2020; Hegazy et al., 2020). Elevated TERRA and telomeric R-loop levels at very short telomeres have been shown to induce telomere HR in human cells (Graf et al., 2017), and telomeric R-loops in ALT cancer cells are important for HR-mediated telomere maintenance (Arora et al., 2014). Surprisingly, TRF2 facilitates telomeric R-loop formation and TRF1 antagonizes this function (Lee et al., 2018), suggesting that the telomeric R-loop-induced telomere DNA damage is not a major threat to human telomere integrity. Telomere proteins in yeasts also suppresses NHEJ and HR: *Saccharomyces cerevisiae* RAP1 suppresses NHEJ (Pardo and Marcand, 2005), and *Schizosaccharomyces pombe* RAP1 appears to suppress HR induced by a high level of telomeric R-loop in cells lacking the telomerase recruitment factor Ccq1 (Hu et al., 2019).

Interestingly, *T. brucei* does not have the NHEJ machinery (Burton et al., 2007), as the *T. brucei* genome lacks the DNA ligase IV that is essential for the NHEJ pathway, and no NHEJ events have been observed in this parasite. Therefore, *T. brucei* telomeres are not threatened by the NHEJ-mediated chromosome end-to-end fusions. However, HR events have been frequently observed at subtelomeric regions, where HR is clearly one of the major pathways of VSG switching (McCulloch et al., 2015). Therefore, telomere HR can be an important instability factor in *T. brucei*. In addition, telomeric R-loop-induced telomere DNA damage can be a great threat to telomere/subtelomere integrity in *T. brucei* and induce subtelomeric HR events (Jehi et al., 2014a; Nanavaty et al., 2017; Briggs et al., 2018; Saha et al., 2021). TERRA has been detected in *T. brucei* (Rudenko and Van der Ploeg, 1989; Damasceno et al., 2017; Nanavaty et al., 2017; Saha et al., 2019, 2021). *T. brucei* TERRA transcription has several unique features (Saha et al., 2021). First, TERRA appears to be transcribed only from the active *VSG*-adjacent telomere, as the polycistronic transcript including the active *VSG* (but not silent *VSG* or a *VSG*-free subtelomere) and TERRA sequences can be detected by RT-PCR (Damasceno et al., 2017; Nanavaty et al., 2017; Saha et al., 2021). Most *T. brucei* cells have 1–3 nuclear TERRA foci, and in cells that have 2–3 TERRA foci (∼39% of G1 and 57–63% S and G2/M cells), frequently only the brightest TERRA focus is co-localized with the telomere (Saha et al., 2021). Second, TERRA is transcribed by RNA Polymerase I in *T. brucei*, as it is not sensitive to α-Amanitin (Rudenko and Van der Ploeg, 1989), and treating cells with an RNA Polymerase I inhibitor, BMH-21, for only 15 min can abolish >92% of TERRA (Saha et al., 2021). The RNA Polymerase I-mediated TERRA transcription is apparently at a very high level and can be better appreciated when *Tb*TRF is depleted, where a single TERRA focus is frequently observed in the nucleus, and the size of the TERRA focus can be nearly as big as the nucleolus (Saha et al., 2021). The single TERRA transcription site presumably also helps to increase local TERRA concentration at the active telomere, which promotes telomeric R-loop formation. Indeed, telomeric R-loops are readily detectable in WT *T. brucei* cells (Nanavaty et al., 2017; Saha et al., 2021). Intriguingly, the active ES-adjacent telomere (but not silent telomeres) frequently experiences large truncations (Bernards et al., 1983), suggesting that TERRA transcription and/or telomeric R-loops formed at the active telomere promote telomere instability. Hence, *T. brucei* telomere faces a great threat from telomeric R-loop-induced telomere/subtelomere DNA damage. Our recent studies further indicate that suppressing the telomeric R-loop level is an important end protection function of *T. brucei* telomere proteins (see below) (Nanavaty et al., 2017; Saha et al., 2021). Furthermore, introducing a DSB at the active *VSG* vicinity induces many more DNA recombination-mediated VSG switching events (Boothroyd et al., 2009; Glover et al., 2013), suggesting that telomeric R-loop-induced telomere and subtelomeric DNA damage can be repaired by HR. A direct link between elevated amount of telomeric R-loop, increased amount of DNA damage at the telomere/subtelomere, and many more *VSG* GC-mediated VSG switchings has been established in *Tb*RAP1-depleted cells, where overexpression of RNaseH1 that specifically degrades RNA in the RNA:DNA hybrid suppresses all three phenotypes (Nanavaty et al., 2017). Therefore, *T. brucei* telomere proteins have an important role to suppress the telomeric R-loop level and telomere HR.

It is important to note that a certain degree of telomere and subtelomere plasticity is beneficial to *T. brucei*, as all *VSG* genes are located at subtelomere regions (Müller et al., 2018), and HR is an important means of VSG diversification and a major pathway of VSG switching (Myler et al., 1984a; McCulloch et al., 2015). Indeed, our studies have shown that telomere and subtelomere instability contributes to increased VSG switching frequencies (Benmerzouga et al., 2013; Jehi et al., 2014a, b; Nanavaty et al., 2017; Afrin et al., 2020a; Saha et al., 2021). In the case of *Tb*TRF and *Tb*RAP1, their roles in suppression of the telomeric R-loop level help maintain telomere and subtelomere integrity and suppress VSG switching frequency (see below). Therefore, these telomere proteins have a delicate job to balance the genome stability and plasticity at *T. brucei* telomeres and subtelomeres.

### Shelterin Homologs in *T. brucei*

In mammalian cells, Shelterin associates with the telomere tightly and plays indispensable roles in telomere end protection and telomere length regulation (de Lange, 2005, 2018). Several Shelterin homologs have been identified in *T. brucei* (Li et al., 2005; Yang et al., 2009; Jehi et al., 2014b). Here I will focus on different mechanisms underlying *Tb*TRF and *Tb*RAP1’s functions when compared to their mammalian and yeast homologs.

#### *T. brucei* TRF vs. Mammalian TRF1/2

**FIGURE 2:**
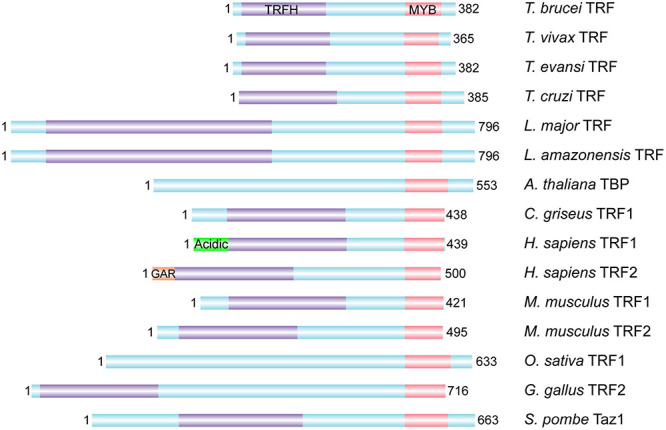
TRF homologs from various organisms. The DNA binding Myb domain, the homodimerization TRFH domain, the N-terminal acidic domain of human TRF1 and basic GAR domain of human TRF2 are marked whenever identified. In addition, *Leishmania amazonensis* TRF has been identified and shown to associate with the telomere (da Silva et al., 2010). *T. brucei*, *Trypanosoma brucei*; *T. vivax*, *Trypanosoma vivax*; *T. evansi*, *Trypanosoma evansi*; *T. cruzi*, *Trypanosoma cruzi*; *L. major*, *Leishmania major*; *L. amazonensis*, *Leishmania amazonensis*; *A. thaliana*, *Arabidopsis thaliana*; *C. griseus*, *Cricetulus griseus*; *H. sapiens*, *Homo sapiens*; *M. musculus*, *Mus musculus*; *O. sativa*, *Oryza sativa*; *G. gallus*, *Gallus gallus*; *S. pombe*, *Schizosaccharomyces pombe*.

*Tb*TRF was identified as the duplex TTAGGG repeat binding factor in *T. brucei* (Figure 2; Li et al., 2005). Its duplex telomere binding activity resides in the C-terminal Myb domain but it does not bind single stranded DNA (Li et al., 2005), which is similar to its mammalian homologs TRF1 and TRF2 (Zhong et al., 1992; Bilaud et al., 1997; Broccoli et al., 1997b). *Tb*TRF associates with the telomere chromatin, and is almost always co-localized with the telomere as shown in telomere FISH combined with *Tb*TRF IF experiments (Li et al., 2005). Mammalian TRF1 and TRF2 both have a TRF Homology (TRFH) domain in the N-terminal half of the protein (Broccoli et al., 1997b), which is responsible for TRF homodimerization (Fairall et al., 2001; Chen et al., 2008). In addition, human TRF1 has an acidic N-terminus (Broccoli et al., 1997a), while TRF2 has a basic N-terminal GAR domain (Broccoli et al., 1997b; Mitchell et al., 2009). *Tb*TRF also has a TRFH domain that mediates homodimerization, although the *Tb*TRFH domain only presents limited sequence and structure homology with its mammalian counterparts (Li et al., 2005).

It is impossible to tell whether *Tb*TRF is more homologous to TRF1 or TRF2 based solely on sequence analysis, as *Tb*TRF lacks an N-terminal domain (Figure 2; Li et al., 2005). Depletion of *Tb*TRF leads to a loss of the telomere 3′ overhang structure (Li et al., 2005), while removal of TRF2 from the telomere results in the same phenotype (van Steensel et al., 1998), indicating that *Tb*TRF and TRF2 have the same function in maintaining the telomere terminal structure. On the other hand, TRF2 facilitates telomeric R-loop formation while TRF1 suppresses this effect (Lee et al., 2018), and *Tb*TRF also suppresses the telomeric R-loop level (Saha et al., 2021), indicating that *Tb*TRF and TRF1 have similar effects on telomeric R-loop. Recently, it has been shown that human TRF2 at the telomere is sufficient and necessary for the T-loop formation (Doksani et al., 2013; Timashev and de Lange, 2020), which in turn suppresses ATM activation (Van Ly et al., 2018). The T-loop structure has been observed in *T. brucei* (Munoz-Jordan et al., 2001). However, it is unknown whether *Tb*TRF is required to establish/maintain the T-loop structure. In addition to suppression of ATM activation, TRF2 also prevents the NHEJ-mediated chromosome end-to-end fusions (Karlseder et al., 1999; Smogorzewska et al., 2002; Celli and de Lange, 2005), and its N-terminal basic domain suppresses HR-mediated telomere recombinations (Wang et al., 2004).

In *T. brucei*, although NHEJ is absent, telomeres and subtelomeres are fragile (Glover et al., 2013), and HR is the major mechanism of VSG switching (Navarro and Cross, 1996; Kim and Cross, 2010; Benmerzouga et al., 2013; Jehi et al., 2014a, b; Nanavaty et al., 2017; da Silva et al., 2018). Therefore, HR is a major DNA damage response pathway in *T. brucei* (Glover et al., 2008) and posts realistic threat to telomere and subtelomere stability. Indeed, in *Tb*TRF-depleted cells, the γH2A level is increased (Saha et al., 2021), where γH2A is the C-terminal phosphorylated H2A that is deposited to the chromatin at DNA damage sites in *T. brucei* (Glover and Horn, 2012). Furthermore, a transient depletion of *Tb*TRF leads to an increased number of VSG switching events, most of which involving the loss of the originally active ES (Jehi et al., 2014a). As expected, *Tb*TRF’s role in maintaining telomere and subtelomere stability requires its telomere DNA binding activity (Jehi et al., 2014a). Unexpectedly, depletion of *Tb*TRF results in an increased amount of TERRA (Saha et al., 2021). *Tb*TRF does not affect TERRA’s half-life (Saha et al., 2021). Rather, a higher level of the polycistronic transcript containing the TERRA sequence and the active *VSG* sequence is detected upon *Tb*TRF depletion, suggesting that *Tb*TRF normally suppresses TERRA transcription. It is possible that *Tb*TRF’s binding to the telomere DNA directly hinders RNA Polymerase I-mediated TERRA transcription. On the other hand, although *Tb*TRF depletion does not derepress silent polycistronic BF *VSG* ESs (Figure 1; Yang et al., 2009), it does derepress subtelomeric monocistronic metacyclic *VSG* ESs (Figure 1; Saha et al., 2021), indicating that *Tb*TRF is likely important for telomeric silencing, but its effect spreads only to a short distance from the telomere. *Tb*TRF’s telomeric silencing function presumably contributes to TERRA suppression. In addition, the amount of telomeric R-loops is increased upon depletion of *Tb*TRF (Saha et al., 2021). Overexpression of RNaseH1 reduces the telomeric R-loop level in *Tb*TRF-depleted cells and the amount of DNA damage, confirming that more telomeric R-loops cause more telomere DNA damage upon *Tb*TRF depletion (Saha et al., 2021). Therefore, these observations indicate that *Tb*TRF helps maintain telomere integrity through suppressing the levels of TERRA and telomeric R-loop (Saha et al., 2021), which is similar to TRF1 but different from TRF2 (Lee et al., 2018).

Depletion of human TRF2 also results in an increased level of TERRA (Porro et al., 2014b). In addition, TRF2 binds TERRA predominantly through its N-terminal GAR domain (Deng et al., 2009b; Mei et al., 2021; Figure 2). *Tb*TRF also has a TERRA binding activity, which surprisingly resides in its Myb domain (Saha et al., 2021). Most interestingly, a *Tb*TRF Myb domain point mutant that loses its telomere DNA binding activity (Jehi et al., 2014a) binds TERRA more strongly (Saha et al., 2021). In addition, *Tb*TRF exhibits a slightly stronger affinity to the duplex telomere DNA than TERRA in *in vitro* competition binding assays (Saha et al., 2021). Therefore, the telomere DNA binding and TERRA-binding activities of *Tb*TRF may have overlapping nucleic acid interaction interfaces, which is clearly different from how human TRF 2 binds TERRA and telomere DNA (Bilaud et al., 1997; Broccoli et al., 1997b; Deng et al., 2009b; Mei et al., 2021).

The fact that *Tb*TRF has both TERRA and a ds(TTAGGG)_*n*_ binding activities provides an additional possible mechanism how *Tb*TRF regulates the level of the telomeric R-loop. Significantly more *Tb*TRF-depleted cells (in G1, S, or G2/M phases) than WT cells have only one TERRA focus (Saha et al., 2021), suggesting that *Tb*TRF helps recruit TERRA away from its transcription site. In this case, the TERRA and ds(TTAGGG)_*n*_ binding activities of *Tb*TRF can help transport TERRA to TTAGGG repeats other than its transcription site, as *Tb*TRF also has a homodimerization function (Li et al., 2005). Although *Tb*TRF can theoretically bind all telomeres, it is expected that the actively transcribed telomere region is mostly free of *Tb*TRF due to the high-level RNA Polymerase I-mediated transcription, similar to the situation in the active ES, which is deprived of nucleosomes (Figueiredo and Cross, 2010; Stanne and Rudenko, 2010). Translocation of TERRA away from its transcription site will effectively limit TERRA accumulation at a single telomere, significantly reducing the chance of telomeric R-loop formation. Therefore, *Tb*TRF may suppress the telomeric R-loop level through suppressing of TERRA transcription and promoting TERRA translocation. On the other hand, human TRF1 and TRF2 have an opposite effect and restrict TERRA’s translocation away from its transcription site (Feretzaki et al., 2020). The key telomeric functions of human TRF1/2 and *Tb*TRF are compared in Table 1.

**TABLE 1:** Summary of key telomere functions of human and *T. brucei* TRF proteins.

**TRF Homolog**	**Key telomeric functions**	**References**
TRF1	Facilitates telomere DNA replication	Sfeir et al., 2009
	Suppresses telomerase-mediated telomere elongation	van Steensel and de Lange, 1997; Smogorzewska et al., 2000
	Suppresses TRF2-mediated telomeric R-loop formation	Lee et al., 2018
	Suppresses *trans*-localization of TERRA	Feretzaki et al., 2020
	Suppresses the TERRA level in pluripotent cells	Marión et al., 2019
TRF2	Maintains the telomere G-overhang structure	van Steensel et al., 1998
	Suppresses NHEJ-mediated chromosome end-to-end fusions	van Steensel et al., 1998; Karlseder et al., 1999; Celli and de Lange, 2005
	Suppresses telomere HR	Wang et al., 2004
	Suppresses telomerase-mediated telomere elongation	Smogorzewska et al., 2000
	Promotes telomeric R-loop formation	Lee et al., 2018
	Facilitates the T-loop structure formation and maintenance	Doksani et al., 2013; Timashev and de Lange, 2020
	Suppresses *trans*-localization of TERRA	Feretzaki et al., 2020
	The GAR domain is essential for binding TERRA	Deng et al., 2009b; Mei et al., 2021
*Tb*TRF	Maintains the telomere G-overhang structure	Li et al., 2005
	Maintains telomere integrity	Saha et al., 2021
	Suppresses DNA recombination at the subtelomere	Jehi et al., 2014a
	Important for short-range telomeric silencing	Saha et al., 2021
	Suppresses the TERRA level	Saha et al., 2021
	Facilitates *trans*-localization of TERRA	Saha et al., 2021

#### RAP1 Homologs in Vertebrates, Yeasts, and *T. brucei*

**FIGURE 3:**
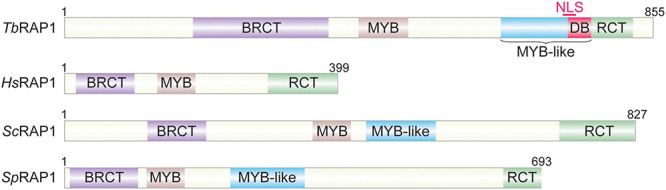
Repressor activator protein 1 (RAP1) homologous. *Tb*, *Trypanosoma brucei*; *Hs*, *Homo sapiens*; *Sc*, *Saccharomyces cerevisiae*; *Sp*, *Schizosaccharomyces pombe*.

*Saccharomyces cerevisiae* RAP1 is the first protein identified to directly bind telomere DNA among yeast and vertebrate telomere proteins (Shore and Nasmyth, 1987; Longtine et al., 1989; Conrad et al., 1990). RAP1 is also one of the most conserved telomere proteins (Figure 3), with its homologs identified in kinetoplastids (Yang et al., 2009), yeasts (Shore and Nasmyth, 1987; Kanoh and Ishikawa, 2001; Wahlin and Cohn, 2002; Yu et al., 2010; Steinberg-Neifach and Lue, 2015), and vertebrates (Li et al., 2000; Tan et al., 2003).

The functions of yeast RAP1 homologs in telomeric silencing and telomere length regulation have been extensively studied (Lustig et al., 1990, 1996; Hardy et al., 1992; Kyrion et al., 1992, 1993; Liu et al., 1994; Cockell et al., 1995; Liu and Lustig, 1996; Marcand et al., 1997; Wotton and Shore, 1997; Kanoh and Ishikawa, 2001). In addition, *Sc*RAP1 prevents NHEJ-mediated telomere end-to-end fusions (Pardo and Marcand, 2005). On the other hand, the functions of mammalian RAP1 homologs have been somewhat hard to define. Initial studies in telomerase positive cancer cell lines indicated that human RAP1 is involved in telomere length regulation (Li et al., 2000; Li and de Lange, 2003). Subsequently, *in vitro* biochemical study and engineered tethering of human RAP1 to the telomere both indicated that RAP1 is capable of suppressing NHEJ at the telomere (Bae and Baumann, 2007; Sarthy et al., 2009). However, TALEN-mediated deletion of the human RAP1 exon 2 in a number of cell lines showed that hRAP1 is not required for telomere length regulation or suppression of telomere end-to-end fusions (Kabir et al., 2014). Recently, it has been shown that human RAP1 is required to inhibit NHEJ-mediated telomere fusions at critically short telomeres (Lototska et al., 2020). On the other hand, conditional knockout of mouse RAP1 or expression of a mouse TRF2 mutant that does not interact with RAP1 (so that RAP1 is not recruited to the telomere) showed that mouse RAP1 coordinates with TRF2 N-terminal basic domain to suppress telomere HR (Sfeir et al., 2010; Rai et al., 2016).

*Trypanosoma brucei* RAP1 was identified as a *Tb*TRF-interacting factor (Yang et al., 2009). *Tb*RAP1 was the first telomere protein that has been shown to be essential for *VSG* monoallelic expression, as depletion of *Tb*RAP1 by RNAi or conditional knockout of *Tb*RAP1 result in derepression of essentially all subtelomeric *VSG* genes in *T. brucei* (Yang et al., 2009; Pandya et al., 2013; Afrin et al., 2020b). The silent ES-linked *VSGs* are derepressed up to several thousand folds, which represents the most dramatic VSG derepression phenotype among *T. brucei* mutants that affect VSG silencing (Figueiredo et al., 2008; Yang et al., 2009; Alsford and Horn, 2012; Tiengwe et al., 2012; Benmerzouga et al., 2013; Kim et al., 2013; Cestari and Stuart, 2015; Glover et al., 2016; Reynolds et al., 2016; Schulz et al., 2016; Briggs et al., 2018, 2019; Faria et al., 2019; Kim, 2019). In addition, depletion of *Tb*RAP1 leads to an increased amount of DNA damage at the telomere and subtelomere and more VSG switching events (Nanavaty et al., 2017; Afrin et al., 2020a, b). Interestingly, depletion of *Tb*RAP1 also leads to elevated levels of TERRA and telomeric R-loops (Nanavaty et al., 2017). Overexpression of an ectopic allele of RNaseH1 in the *Tb*RAP1-depleted cells suppresses the increased amount of telomeric R-loop and DNA damage and the increased VSG switching frequency phenotypes, while the TERRA level in these cells is still higher than that in WT cells (Nanavaty et al., 2017). Therefore, the function of *Tb*RAP1 in maintaining telomere/subtelomere integrity relies on its repressive effects on TERRA and telomeric R-loop. The TERRA molecules in *Tb*RAP1-depleted cells are much longer than that in WT cells (Nanavaty et al., 2017), suggesting that *Tb*RAP1 can block the transcription elongation of RNA Polymerase I along the telomere by associating with the telomere chromatin. These observations indicate that *Tb*RAP1 suppresses telomeric and subtelomeric HR by suppression of the telomeric R-loop level. A recent study in fission yeast suggests that *Sp*RAP1 has a similar suppressive effect on telomeric R-loop and telomeric HR (Hu et al., 2019). Therefore, the function of RAP1 homologs in suppressing telomere HR appears to be conserved, although there is no direct evidence showing that mammalian RAP1 suppresses the telomeric R-loop level.

All telomere functions of RAP1 homologs depend on their telomere association. However, RAP1 homologs achieve this goal through different mechanisms. *Sc*RAP1 has both a Myb domain and a Myb-Like motif in the central region of the protein (Figure 3), which were confirmed to contain the duplex DNA binding activity (Konig et al., 1996), recognizing a consensus sequence 5′ ACACCCAYACAYY 3′ (where Y represents a pyrimidine) (Graham and Chambers, 1994). However, this sequence-specific duplex DNA binding activity was only identified in budding yeast RAP1 homologs (Konig et al., 1996; Rhodin et al., 2006; Yu et al., 2010). Human RAP1 is only recruited to the telomere through its interaction with TRF2, while itself does not directly bind the telomere DNA (Li et al., 2000; Loayza and de Lange, 2003). Similarly, *Sp*RAP1 is also recruited to the telomere through its interaction with TAZ1 (Kanoh and Ishikawa, 2001), the duplex telomere DNA binding factor in *S. pombe* and the functional homolog of mammalian TRF1/2 (Cooper et al., 1997; Li et al., 2000). It was originally hypothesized that *Tb*RAP1 was also recruited to the telomere via its interaction with *Tb*TRF (Yang et al., 2009). However, *Tb*RAP1 still associates with the telomere chromatin in *Tb*TRF-depleted cells (Afrin et al., 2020a), and *Tb*RAP1’s telomere association does not require its Myb domain (Yang et al., 2009; Afrin et al., 2020a), even though Myb motifs frequently have DNA binding activities (Ogata et al., 1994). Rather, we recently identified both dsDNA and ssDNA binding activities in *Tb*RAP1 (Afrin et al., 2020a). Both activities are electrostatic-based and require a positively charged _737_RKRRR_741_ patch that overlaps with the nuclear localization signal (NLS) in the Myb-Like domain of *Tb*RAP1 (Figure 3; Yang et al., 2009; Afrin et al., 2020a). Proteomic studies detected phosphorylated S742 and S744 residues in *T. brucei* cells (Nett et al., 2009; Urbaniak et al., 2013). Interestingly, the phospho-mimic S742DS744D mutation of *Tb*RAP1 disrupts most of its dsDNA binding activity but retains most of its ssDNA binding activity (Afrin et al., 2020a). In addition, *Tb*RAP1-S742DS744D is no longer associated with the telomere chromatin while *Tb*RAP1-S742AS744A is still located at the telomere, suggesting that phosphorylation of the two _737_RKRRR_741_-adjacent S residues can remove *Tb*RAP1 from the telomere (Afrin et al., 2020a). Furthermore, VSG silencing and telomere integrity are disrupted in all *Tb*RAP1 mutants that do not associate with the telomere chromatin, further suggesting that phosphorylation of the two S residues can regulate VSG silencing and telomere integrity in *T. brucei* cells (Afrin et al., 2020a). The *Tb*RAP1’s DNA binding activities are quite different from that of *Sc*RAP1. *Sc*RAP1’s duplex DNA binding activity is sequence specific and depends on its Myb and Myb-Like domains (Konig et al., 1996). So far no protein modification has been shown to regulate *Sc*RAP1’s DNA binding activity. On the other hand, *Tb*RAP1 has both dsDNA and ssDNA binding activities, which are sequence non-specific, and phospho-mimicking mutation of S742 and S744 can disrupt its dsDNA binding activity (Afrin et al., 2020a). Therefore, although the main functions of RAP1 homologs at the telomere are conserved from kinetoplastids to mammals, the underlying mechanisms are clearly different among different RAP1 homologs. The key telomeric functions of human, yeast, and *T. brucei* RAP1 homologs are compared in Table 2.

**TABLE 2:** Summary of key telomeric functions of RAP1 homologs.

**RAP1 homologs**	**Key telomeric functions**	**References**
Mammalian RAP1	Suppresses telomere HR (mRAP1)	Rai et al., 2016; Sfeir et al., 2010
	Suppresses NHEJ-mediated telomere end fusions at critically short telomeres (hRAP1)	Lototska et al., 2020
	Does not bind telomere DNA directly *in vivo* (hRAP1)	Li et al., 2000; Loayza and de Lange, 2003
*Sc*RAP1	Has a sequence-specific dsDNA binding activity	Graham and Chambers, 1994; Konig et al., 1996
	Nucleates the telomeric heterochromatic structure, essential for telomeric silencing	Hardy et al., 1992; Kyrion et al., 1993; Liu et al., 1994; Cockell et al., 1995; Liu and Lustig, 1996
	Suppresses NHEJ-mediated chromosome end-to-end fusions	Pardo and Marcand, 2005
	Suppresses telomerase-mediated telomere elongation	Lustig et al., 1990; Kyrion et al., 1992; Marcand et al., 1997; Wotton and Shore, 1997
	Suppresses the TERRA level	Iglesias et al., 2011
*Sp*RAP1	Does not bind telomere DNA directly *in vivo*	Kanoh and Ishikawa, 2001
	Suppresses telomerase-mediated telomere elongation	Kanoh and Ishikawa, 2001
	Essential for telomeric silencing	Kanoh and Ishikawa, 2001
	Maintains genome stability	Irie et al., 2019
	Suppresses telomeric transcripts	Bah et al., 2012
	Suppresses telomeric HR	Hu et al., 2019
*Tb*RAP1	Essential for telomeric silencing	Yang et al., 2009; Pandya et al., 2013; Afrin et al., 2020a, b
	Essential for monoallelic *VSG* expression	Yang et al., 2009; Afrin et al., 2020a, b
	Maintains telomere and subtelomere genome integrity	Nanavaty et al., 2017; Afrin et al., 2020a
	Suppresses HR at the subtelomere	Nanavaty et al., 2017
	Suppresses the TERRA level	Nanavaty et al., 2017
	Suppresses the telomeric R-loop level	Nanavaty et al., 2017
	Has both electrostatic-based, sequence-non-specific ssDNA and dsDNA binding activities	Afrin et al., 2020a
	DNA binding motif overlaps with nuclear localization signal	Afrin et al., 2020a

The fact that *Tb*TRF and *Tb*RAP1 are essential for maintaining telomere integrity and stability indicates that these telomere proteins have conserved essential functions as their yeast and mammalian homologs. However, recent findings indicate that the underlying mechanisms of how *Tb*TRF and *Tb*RAP1 achieve their goals are quite different from those in human and yeast cells. First, *T. brucei* does not have the NHEJ machinery. Therefore, neither *Tb*TRF nor *Tb*RAP1 needs to suppress any NHEJ-mediated telomere fusions. Second, telomeric R-loops have been shown to be an important factor contributing to telomere damage if not controlled at a WT level in *T. brucei* (Nanavaty et al., 2017; Briggs et al., 2018; Saha et al., 2021). While both *Tb*TRF and *Tb*RAP1 suppress the level of telomeric R-loop (Nanavaty et al., 2017; Saha et al., 2021), whether mammalian RAP1 homologs have the same function is unknown, and human TRF2 actually stimulates the formation of telomeric R-loop, which is antagonized by human TRF1 (Lee et al., 2018). The unique features of *Tb*RAP1 (such as its DNA binding activities) and *Tb*TRF (such as its Myb-mediated TERRA binding activity) telomere functions can be targeted as a means to eliminate the parasites from its mammalian host. In addition, *Tb*RAP1 and *Tb*TRF only have limited sequence homology with their mammalian counterparts in their functional domains (Li et al., 2005; Yang et al., 2009), making it more feasible to specifically target the parasite telomere proteins without affecting their mammalian homologs.

### Subtelomere Plasticity Benefits Antigenic Variation

As discussed earlier, telomere integrity and stability is important for genome integrity. Subtelomere stability is also important for organism health and fitness. In humans, unstable subtelomeres are frequently associated with various diseases. For example, reduced copy number of polymorphic macrosatellite repeat D4Z4 at chromosome 4q subtelomere has long been associated with facioscapulohumeral muscular dystrophy (FSHD) (van der Maarel et al., 2007; Daxinger et al., 2015). Submicroscopic deletion of subtelomeric 6p25 has been recognized as a clinically identifiable syndrome (DeScipio, 2007), and deletion of the *EHMT1* gene at the chromosome 9q subtelomere leads to the Kleefstra syndrome (Stewart and Kleefstra, 2007; Bonati et al., 2019). A recent study further indicates that 3–16% of syndromic intellectual disability cases are caused by cryptic subtelomeric abnormalities (Soares et al., 2019). Subtelomere integrity and stability is also important in *T. brucei*, as *VSG* is essential (Sheader et al., 2005), and all *VSG* genes are located at subtelomeres (de Lange and Borst, 1982; Hertz-Fowler et al., 2008; Müller et al., 2018). DSBs in the active *VSG* gene vicinity are generally poorly tolerated: introducing an artificial DSB (an I-SceI cut) within or near the active *VSG* gene leads to death in more than 80% of the cells (Glover et al., 2013). Inefficient repair of the I-SceI cut due to continued I-SceI expression may contribute to the catastrophic consequence. Nevertheless, the location of the damage site appears to be a critical factor, as inducing the same I-SceI cut in a silent ES is much better tolerated (Glover et al., 2013). However, maintaining subtelomere stability can be challenging. Subtelomeres often consist of duplicated sequence blocks near the ends of multiple chromosomes and are highly dynamic with very heterogeneous sequences, sizes, and copy numbers (Pryde et al., 1997; Mefford and Trask, 2002; Li, 2012). Increased rates of sister chromatid exchange have been observed at human chromosome ends by cytological studies (Rudd et al., 2007), and human subtelomeres are hot spots of interchromosomal recombination and segmental duplications (Linardopoulou et al., 2005). High polymorphism in the subtelomere is frequently observed among different chromosome ends and individuals in humans (Ambrosini et al., 2007; Young et al., 2017), yeast (Pryde et al., 1997; Quispe et al., 2017), fly (Anderson et al., 2008), plant (Kuo et al., 2006), and fungal pathogens (Farman, 2007; Schmid-Siegert et al., 2017). Similarly, it has been shown that *T. brucei* subtelomere is a fragile site (Glover et al., 2013). *T. brucei* homologous megabase chromosome pairs often differ greatly in size (Melville et al., 2000) due to different sizes of subtelomeric ESs and *VSG* gene arrays, telomere, and repetitive chromosomal regions (Melville et al., 1999). In fact, two-thirds of the size polymorphisms are due to variations in subtelomeric regions, while chromosomal core regions, containing all essential genes, are relatively stable (Callejas et al., 2006). Therefore, maintaining subtelomere stability is important yet challenging.

On the other hand, subtelomere plasticity can be beneficial to the organism to a certain extent. For example, some human *olfactory receptor* (*OR*) genes encoding olfactory receptors are located at subtelomeres, and changes in subtelomere regions may contribute to the diversity of the *OR* gene family (Trask et al., 1998). Subtelomeres also have important functions for microbial pathogens, where genes with roles in niche adaptation are frequently enriched (Underwood et al., 1996; Zhang et al., 1997; De Las Penas et al., 2003; Farman, 2007). Subtelomeric plasticity and relative frequent subtelomere recombination in these microbial pathogens is expected to help increase the diversity of their major surface antigen and enhance the effectiveness of evading host immune responses. As described above, in *T. brucei*, removal of telomere proteins (such as *Tb*TRF, *Tb*TIF2–a *Tb*TRF-interacting factor and the human TIN2 homolog, and *Tb*RAP1) leads to telomere instability and increased VSG switching rates (Jehi et al., 2014a, b; Nanavaty et al., 2017; Afrin et al., 2020a). Subtelomere instability has also been frequently observed in *P. falciparum* that causes malaria in humans. At the erythrocyte stage, *P. falciparum* infects the host red blood cells (RBCs) and expresses *Pf*EMP1 on the host RBC surface, which is important for adhering the infected RBCs to the endothelial lining of host blood vessels so that the infected RBCs will not be eliminated by the host immune system (Hviid and Jensen, 2015; Wahlgren et al., 2017). Additional parasite proteins including RIFIN and STEVOR are also expressed on host RBC membrane, facilitating interaction of parasite-infected RBCs and other host cells (Wahlgren et al., 2017). The *var*, *rif*, and *stevor* gene families that encode PfEMP1, RIFIN, and STEVOR, respectively, are mostly located at subtelomeric regions (Rubio et al., 1996; Cheng et al., 1998). *P. falciparum* regularly switches to express different *var*, *rif*, and *stevor* genes to evade the host immune attack (Wahlgren et al., 2017). In addition, *var* gene expression is strictly monoallelic (Voss et al., 2006). Importantly, recent studies showed that *P. falciparum* subtelomeres frequently have HR events that contributes to divergence of *var* gene families (Calhoun et al., 2017), and this subtelomere plasticity is enhanced when a DSB is introduced at the vicinity (Calhoun et al., 2017; Zhang et al., 2019). In the pathogenic yeast *Pneumocystis jirovecii* that causes pneumonia in immunodeficient patients, genes encoding its major surface antigen, MSG, are also located at subtelomeric loci (Keely et al., 2005) and are expressed in a monoallelic fashion (Kutty et al., 2001). There is only one subtelomeric *MSG* ES (Kutty et al., 2001), and antigenic variation is achieved through recombining a silent *MSG* gene into the active ES and creating novel mosaic *MSG* genes though recombination (Stringer, 2007; Kutty et al., 2008; Schmid-Siegert et al., 2017). Even in non-pathogenic microbial organisms, subtelomere plasticity can be beneficial for the organism to better adapt to their living environment. In *K. lactis*, genes encoding β-galactosidase are located at subtelomeres, and variation in these genes allows yeast to better cope with different nutrition (Mason and McEachern, 2018). Interestingly, it has been observed that mild telomere dysfunction that does not induce global genome instability leads to an increased variation of the subtelomere β-galactosidase-coding genes, while severe telomere dysfunction causes complete deletion of these genes (Mason and McEachern, 2018). Therefore, telomere and subtelomere plasticity to a certain extent may not be deleterious but even beneficial to improve the organism’s adaptation to various environmental growth conditions.

### Strategies of Tolerating Mild Telomere/Subtelomere Damage in *T. brucei*

If telomere/subtelomere plasticity and mild telomere damage is beneficial to *T. brucei*, the parasite may have evolved ways to encourage it. Indeed, *T. brucei* does not appear to have a stringent DNA damage surveillance mechanism. The initial hint comes from the study where an I-SceI site was targeted to the junction of telomere and sub-telomere downstream a silent ES in telomerase null cells (Glover et al., 2007). After induction of the I-SceI endonuclease, the marked telomere and the upstream silent *VSG* gene were lost, yet the cells did not go into cell cycle arrest (Glover et al., 2007). Subsequently, it was shown that as short as 40 bp of telomere DNA downstream of a silent ES can be stably maintained in a telomerase-independent manner without iliciting cell cycle arrest (Dreesen and Cross, 2006). In addition, DSBs near silent *VSG*s are much better tolerated than those in the active *VSG* vicinity (Glover and Horn, 2014). Similarly, a single induced I-SCE I cut in *T. brucei* genome failed to activate cell cycle checkpoint (Glover et al., 2019). Therefore, as long as the active *VSG* gene (and essential genes at chromosome core regions) is not damaged and VSG synthesis is normal, individual DSB in *T. brucei* genome, particularly those at telomere/subtelomere vicinity is well-tolerated.

On the other hand, *T. brucei* does have cell cycle arrest mechanisms in response to telomere defects. For example, depletion of *Tb*TRF leads to an acute G2/M cell cycle arrest (Li et al., 2005), while depletion of *Tb*RAP1 results in cell growth arrest with a decrease in the S phase population and an increase in the G2/M population (Yang et al., 2009). Interestingly, depletion of *Tb*TRF caused an increased amount of telomere DNA damage (Saha et al., 2021) and depletion of *Tb*RAP1 results in an increased amount of both telomere and subtelomere DNA damage (Nanavaty et al., 2017), and it is expected that the damage occurs at multiple chromosome ends rather than at a single telomere. Hence, a much higher level of DNA damage occurs in *Tb*TRF- and *Tb*RAP1-depleted cells than a single I-SceI cut. Therefore, *T. brucei* appears to be able to tolerate a very small amount of DNA damage, but still guard against DNA damage of a global scale. Presumably, this will allow more genome plasticity, particularly at the subtelomere regions to facilitate a more effective antigenic variation.

It is interesting to note that *T. brucei* does not have the NHEJ machinery (Burton et al., 2007). This helps avoid deleterious chromosome end fusion products that frequently result from telomere defects in human and yeast cells. Several telomere proteins have been shown to suppress subtelomere HR events (Jehi et al., 2014a, b, 2016; Nanavaty et al., 2017). Therefore, mild telomere protein defects, if tolerated, can enhance antigenic variation by allowing more subtelomere HR events. In fact, we have recently identified *Tb*RAP1-S742AS744A and *Tb*RAP1-S265AS586AS742AS744AT752A mutants that only exhibit a very mild growth defect but increase VSG switching rates (Afrin et al., 2020a). The lack of NHEJ machinery likely helps to achieve this middle ground where mild telomere protein defects and subtly increased subtelomere plasticity can enhance antigenic variation without affecting global genome stability.

## Conclusion Remarks

Although the telomere complex is essential for genomic integrity and chromosome stability in all eukaryotic cells studied, recent discoveries indicate that the detailed mechanisms of the “chromosome end protection” can have different features in different organisms. First, the telomere proteins can face different threats that cause genome instability. Hence, studying telomere biology in pathogenic kinetoplastids yields invaluable information on how telomere proteins suppress telomeric and subtelomeric DNA recombination events, which represent a minor pathway compared to NHEJ in human and yeast cells. Second, recent findings illustrate that telomere protein homologs in different organisms can achieve the same goals using distinct mechanisms, which sheds light on telomere protein evolution and provides potential targets for future development of anti-parasite agents. Third, a better understanding of the balance between stability and plasticity at the telomere and subtelomere in pathogenic eukaryotic micro-organisms will help us to better appreciate how eukaryotic cells adapt to different living conditions and evolve to better survive the environment.

## Author Contributions

The author confirms being the sole contributor of this work and has approved it for publication.

## Conflict of Interest

The author declares that the research was conducted in the absence of any commercial or financial relationships that could be construed as a potential conflict of interest.
